# Attitudes on Methadone Utilization in the Emergency Department: A Physician Cross-sectional Study

**DOI:** 10.5811/westjem.2022.2.54681

**Published:** 2022-04-04

**Authors:** Jessica Heil, Valerie S. Ganetsky, Matthew S. Salzman, Krystal Hunter, Kaitlan E. Baston, Gerard Carroll, Eric Ketcham, Rachel Haroz

**Affiliations:** * Cooper University Health Care, Cooper Research Institute, Camden, New Jersey; † Cooper University Health Care, Center for Healing, Division of Addiction Medicine, Camden, New Jersey; ‡ Cooper University Health Care, Department of Emergency Medicine, Division of Addiction Medicine and Medical Toxicology, Camden, New Jersey; § Presbyterian Healthcare System, Departments of Emergency Medicine and Behavioral Health, Albuquerque, New Mexico

## Abstract

**Introduction:**

Like buprenorphine, methadone is a life-saving medication that can be initiated in the emergency department (ED) to treat patients with an opioid use disorder (OUD). The purpose of this study was to better understand the attitudes of emergency physicians (EP) on offering methadone compared to buprenorphine to patients with OUD in the ED.

**Methods:**

We distributed a perception survey to emergency physicians through a national professional network.

**Results:**

In this study, the response rate was 18.4% (N = 141), with nearly 70% of the EPs having ordered either buprenorphine or methadone. 75% of EPs strongly or somewhat agreed that buprenorphine was an appropriate treatment for opioid withdrawal and craving, while only 28% agreed that methadone was an appropriate treatment. The perceived barriers to using buprenorphine and methadone in the ED were similar.

**Conclusion:**

It is essential to create interventions for EPs to overcome stigma and barriers to methadone initiation in the ED for patients with opioid use disorder. Doing so will offer additional opportunities and pathways for initiation of multiple effective medications for OUD in the ED. Subsequent outpatient treatment linkage may lead to improved treatment retention and decreased morbidity and mortality from ongoing use.

## INTRODUCTION

The opioid epidemic in the United States (US) continues to be a major public health crisis, claiming lives at an alarming rate. In 2019 there were more than 70,000 drug overdose deaths in the US. Of these fatalities, nearly 70% involved opioids.^1^ In 2020 overall drug overdose mortality increased by nearly 30% from 2019.^2^ In addition to the substantial human toll of the opioid epidemic, the associated healthcare, criminal justice, and other societal costs were estimated to be almost $820 billion in 2019.^3^ The economic and human loss is devastating in light of available Food and Drug Administration-approved, evidence-based medications for opioid use disorder (MOUD), including buprenorphine and methadone. However, over 70% of patients with opioid use disorder (OUD) are not receiving these treatments.^4^

The emergency department (ED) remains the safety net and point of entry into the healthcare system for many patients struggling with substance use disorders (SUD). Visits to the ED for opioid overdoses continue to increase. Between 1999–2012, opioid-related encounters in US EDs increased by 170%.^5^ Mortality rates after ED visits for nonfatal opioid overdoses are high; greater than 5% of patients die within one year, with the highest risk period being the first month post-overdose.^6^ As the ED is often the only point of entry into the healthcare system for patients with OUD, there is a tremendous opportunity to reduce treatment gaps through ED-based MOUD initiation and early referral to long-term treatment. 7

Buprenorphine and methadone are both evidence-based treatments for OUD that effectively treat opioid withdrawal symptoms, decrease illicit opioid use, and reduce opioid overdose-related mortality.^8^–^11^ Buprenorphine, a partial μ-opioid agonist with high receptor affinity, is the most common form of MOUD used to initiate treatment in the ED. 12 Buprenorphine initiation in the ED and referral to outpatient treatment has been shown to be safe and effective, with increased addiction-treatment engagement at 30 days after discharge compared to brief intervention with referral or referral-only interventions.^13^ Although adoption of this practice has been slow, recently published surveys found substantial support for ED-initiated buprenorphine among emergency physicians (EP).^14^–^16^ Likewise, the American College of Emergency Physicians (ACEP) and the American Academy of Emergency Medicine published position statements recommending that EPs initiate patients on buprenorphine in the ED and offer linkage to outpatient follow-up treatment.^12^,^17^

Methadone, a full μ-opioid agonist, is the most studied MOUD and has been used for over 50 years to treat OUD.^18^ The number of facilities offering buprenorphine increased by an average of 9% annually between 2009–2018, while the number of facilities that offered methadone only increased by an average of 2% per year.^19^ Although methadone is associated with higher treatment retention rates than buprenorphine, methadone carries a considerable social stigma among clinicians because patients treated with methadone are perceived to be more medically complex and therefore difficult to treat.^11^,^20^ In addition to stigma, access to methadone is more difficult because it must be obtained daily and in person at an opioid treatment program (OTP), while buprenorphine can be prescribed in an office-based setting by an X-waivered physician.^18^

Whereas ED-based buprenorphine initiation with long-term treatment linkage has been previously described, data is lacking on methadone initiation in the ED.^13^ To date, there is only one study evaluating low-dose intramuscular administration of methadone to treat opioid withdrawal syndrome in the ED.^21^ While buprenorphine is more commonly initiated in the ED, methadone is a life-saving alternative treatment option that may be preferred by patients who have not been successful with buprenorphine in managing their OUD. 22 Creating more opportunities and pathways for initiation of multiple effective forms of MOUD in the ED and subsequent outpatient treatment linkage may lead to improved treatment retention, as well as decreased morbidity and mortality associated with ongoing opioid use. Thus, it is vital to get EPs’ perspectives on ED-based methadone initiation for patients with OUD.

**Population Health Research Capsule**

What do we already know about this issue?

*Buprenorphine and methadone are effective medications to treat opioid use disorder (OUD), but only buprenorphine has been studied in the Emergency Department (ED).*

What was the research question?

*Do emergency physicians (EP) prefer to treat OUD in the ED with buprenorphine over methadone?*

What was the major finding of the study?

*Seventy-five percent of EP’s agreed that buprenorphine was an appropriate treatment for OUD, while only 28% agreed that methadone was an appropriate treatment.*

How does this improve population health?

*Using this data, interventions could be created to increase methadone initiation in the ED, thus creating more treatment pathways for people with OUD.*

The purpose of this study was to better understand the attitudes of EPs on offering methadone compared to buprenorphine to patients with OUD in the ED. We tested the hypothesis that EP survey respondents would express preferences for buprenorphine over methadone.

## METHODS

### Overview

We conducted a cross-sectional survey of EPs to quantify their opinions, using a five-point Likert scale, regarding the prescribing of buprenorphine and the dosing of methadone in the ED. We did this by creating a survey based on similar work that measured EPs’ willingness to initiate buprenorphine in the ED.^14^ We sent our survey via email to all members of the ACEP Emergency Medicine Practice Research Network (EMPRN), which during the past several years has had approximately 700 to 1200 members. Our institutional review board approved this study and waived informed consent.

### Subjects

Members of ACEP EMPRN are board-certified EPs who represent a cross-section of EPs in the US. Members of EMPRN are asked to participate in surveys distributed via email several times per year.

### Data Collection

The survey was initially emailed to ACEP EMPRN members in early March 2021. Reminder emails to complete the survey were subsequently sent in late March and April 2021. Participation in surveys was voluntary and respondents were not required to answer all questions. Data was collected and stored in the secure ACEP member communication and management platform. To avoid social desirability bias, all participants were given a unique participant ID, and survey results were de-identified prior to being returned to the investigators.

### Survey

Our survey instrument was based on a previously published survey describing physician attitudes on buprenorphine induction in the ED.^14^ We adapted questions specifically focusing on MOUD and excluded any questions about non-opioid treatment of withdrawal symptoms or emergency naloxone prescribing at discharge. We also added a question about referring patients to outpatient clinics that provide MOUD as it applied to the role of the emergency clinician in addressing opioid use. Additionally, the original survey did not include questions on methadone initiation in the ED, and so these were added to the instrument. We collected basic demographics, including primary practice location (urban, rural, or suburban), primary practice region (Northeast, South, Midwest, West), type of healthcare system (community, academic, or federal government hospital), and years out of training. Additionally, we asked whether the physician had obtained their X-waiver, whether they had ever ordered buprenorphine or methadone while working in the ED, and whether their department offered a “warm handoff” or a bridge program to outpatient treatment at discharge for ongoing methadone or buprenorphine treatment.

To compare EPs’ attitudes between buprenorphine and methadone, we asked the same perception questions about both forms of MOUD on a five-point Likert scale. Then we asked respondents to rank perceived barriers to prescribing buprenorphine or dosing methadone in the ED. To prevent participants from completing the survey multiple times, each member of EMPRN and their email addresses were assigned a unique participant ID. If there were multiple entries under the same ID, this was reflected in the data received from EMPRN. Additionally, before every reminder email, the mailing list was edited to reflect who had already responded, and the reminder email was only sent to members who had not responded.

The survey instrument is provided in the “Supplementary Materials” section under Appendix.

### Data Analysis

We used descriptive analysis to summarize response frequency and percentage as well as compare responses between buprenorphine and methadone. Using chi-square tests, we evaluated responses to questions about whether the physician had ever ordered buprenorphine or methadone while working in the ED, had obtained X-waiver training, and whether their department offered a “warm handoff” or a bridge program to outpatient treatment at discharge based on primary practice location. The frequency of the ranked barriers to prescribing buprenorphine or dosing methadone in the ED was descriptively compared. Lastly, we grouped participants based on the presence of a warm handoff or bridge program and measured the likelihood of prescribing either MOUD as well as the highest perceived barriers to prescribing buprenorphine and dosing methadone using chi-square testing.

The response rate was calculated using the number of unique emails in the EMPRN database and the number of physicians who either partially or completely finished the survey. Respondents were not required to answer each question to participate in this study. Therefore, we are reporting the number of survey responses generated for each question. Since this is a descriptive study, a sample size was not needed to determine statistical significance.

## RESULTS

A total of 141 EPs either completed all or some of the survey, with a response rate of 18.4% (141/765). The majority of participants were male (80.9%), White (82.5%), and had a mean age of about 53 years (Table 1). Thirty-four percent of respondents were located in the southern US. The largest group of participants reported their primary practice location as urban (44.3%), and the majority were practicing within a community setting (63.8%).

The majority of EPs reported ordering either buprenorphine, methadone, or both buprenorphine and methadone (69.5%) while working in the ED (Table 1). Further, about 38% of respondents reported having obtained their X-waiver to prescribe buprenorphine. The majority of participants (57.4%) reported that their department did not offer a “warm handoff” or a bridge program to outpatient treatment at discharge for ongoing methadone or buprenorphine treatment.

**Figure 1:**
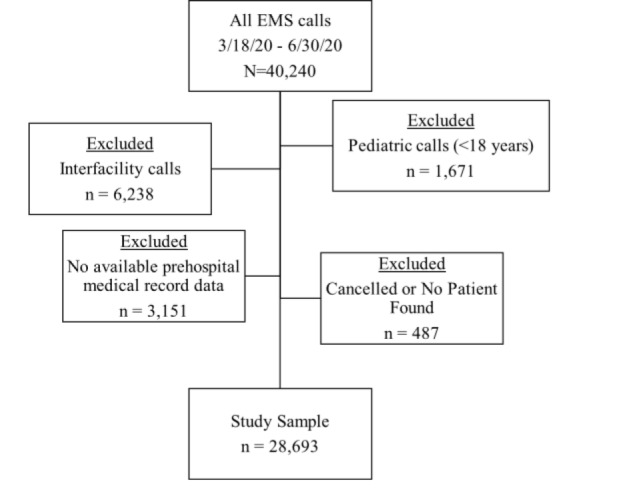
Perceptions questions on buprenorphine and methadone.

Overall, participants had more favorable opinions of using buprenorphine to treat OUD in the ED than methadone. The majority of participants (75%) strongly or somewhat agreed that emergency clinicians should offer buprenorphine to help control the symptoms of opioid withdrawal and craving (Figure 1). In contrast, only about 28% of respondents strongly or somewhat agreed that EPs should offer methadone. This pattern continued, as 95% of participants strongly or somewhat agreed that they would refer patients with OUD to a clinic that provides buprenorphine, but only 63.6% strongly or somewhat agreed that they would refer patients to a methadone clinic. While nearly 88% of respondents stated that they strongly or somewhat agreed with the statement “If my ED had a structured program, I would be comfortable starting buprenorphine for patients who are continuing it after discharge for the purpose of entering treatment,” only about 45% of respondents strongly or somewhat agreed when asked the same question about methadone. When asked whether they were concerned about patients returning to the ED for refills of buprenorphine or methadone, 34% and 46.1% strongly agreed, respectively (Figure 1). Lastly, 41% of physicians strongly or somewhat agreed with the statement, “Initiating patients on methadone is not within the scope of an [EP’s] practice.”

There was a statistically significant difference between primary practice location and whether EPs had ever ordered either MOUD (P<0.05). There was not a statistically significant difference between primary practice location and whether the physician’s ED offered a “warm handoff” or bridge program to outpatient buprenorphine or methadone treatment at discharge (P = 0.15), or whether the physician had completed X-waiver training (P = 0.08) (Table 2).

**Figure 2:**
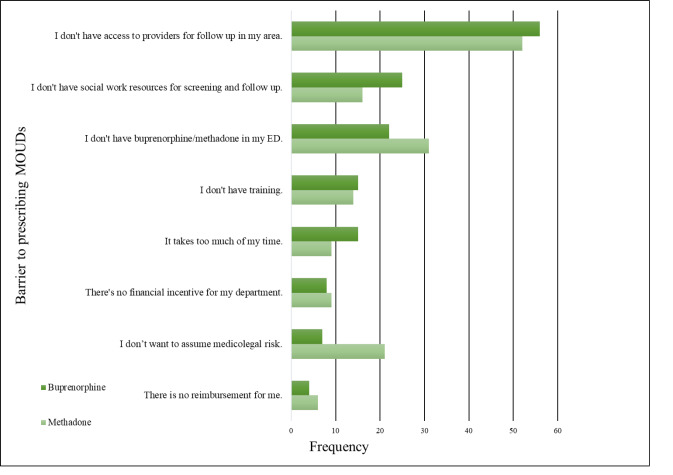
Highest perceived barrier by frequency for prescribing methadone or buprenorphine. *MOUD*, medication for opioid use disorder; *ED*, emergency department.

Emergency physicians reported similar barriers to treating patients in the ED with either buprenorphine or methadone. The two most frequently reported barriers to treating patients with either MOUD were “I don’t have access to providers for follow-up in my area,” and “I don’t have social work resources for screening and follow-up” (Figure 2). Also of note, the responses, “There’s no financial incentive for my department” and “There is no reimbursement for me” were both infrequently reported as barriers to treating with either buprenorphine or methadone.

Next, we grouped participants on whether they had a bridge for either MOUD and measured the highest perceived barriers to prescribing buprenorphine and dosing methadone. One physician reported having a bridge program set up for only methadone dosing. We found that lack of social work resources for screening was a statistically significant barrier for physicians who did not have a bridge for either MOUD (Table 3). For physicians who lacked a bridge, another significant barrier to prescribing buprenorphine was not having buprenorphine in their ED. Physicians with no bridge program reported that their highest perceived barrier to dosing methadone in the ED was lack of training and not having access to OUD experts for follow-up in their area. We also found that having a bridge present in the physician’s ED facilitated the prescribing of MOUD (Table 3).

## DISCUSSION

This study compared EPs’ perceptions of using buprenorphine and methadone in the ED and barriers to the use of these medications to treat OUD in the ED. Overall, this study suggests that EPs prefer to use buprenorphine over methadone. Further, although EPs had a more favorable view of using buprenorphine in the ED than methadone, the most significant barriers to using these medications were similar. In this study, 75% of EPs strongly or somewhat agreed that buprenorphine was an appropriate treatment for opioid withdrawal and craving, while only 28% agreed that methadone was an appropriate treatment. When considering ED referrals, 95% of EPs strongly or somewhat agreed that they would be willing to refer patients to a clinic offering buprenorphine. Only 64% strongly or somewhat agreed that they would refer to a methadone clinic. Even when presented with a structured program for follow-up, only 45% of EPs somewhat or strongly agreed that they felt comfortable initiating methadone in the ED compared to 88% for buprenorphine. Additionally, 41% of physicians did not feel that initiating methadone fell within their scope of practice.

When we grouped our sample by whether a physician had a bridge or not, the highest barrier to prescribing buprenorphine or dosing methadone was a lack of social work resources for screening. Additionally, we found that having a bridge in place helped facilitate the use of MOUD. Both findings are consistent with the literature that states when an ED’s MOUD program includes a follow-up protocol (which could include social workers), physicians feel more comfortable using MOUD to treat OUD in the ED.^23^

Buprenorphine initiation in the ED increases engagement in treatment, decreases illicit opioid use, and has shown to reduce healthcare-related costs due to SUDs.^24^ Long-term outcomes for patients receiving either buprenorphine or methadone include reductions in mortality, opioid use, and opioid-related, acute care utilization.^9^,^10^,^25^ The expansive literature supporting methadone treatment suggests that it may lead to similar, if not better, outcomes than buprenorphine for patients struggling with OUD.^11^,^25^ In contrast to buprenorphine, methadone offers the significant advantage that the patient does not need to experience withdrawal prior to initiation of treatment.^26^,^27^ Drawbacks to ED methadone initiation include its complex pharmacology and adverse effect profile. Rapid initiation can lead to central nervous system depression and respiratory compromise, multiple drug-drug interactions exist, and QTc prolongation has been associated with fatal cardiac events.^18^ The safety profile is better for buprenorphine than methadone, but a single, low dose of methadone 20–40 milligrams is often sufficient to treat opioid withdrawal symptoms with few risks.^12^,^18^

Although methadone has been the mainstay of OUD treatment since the 1970s, ED initiation and treatment have not been incorporated into common practice for OUD, despite clear evidence of efficacy. 28 Since the ED will continue to serve as a critical access point for patients with OUD, adding methadone to an emergency clinician’s toolkit to treat OUD may present a valuable opportunity to reduce treatment gaps through MOUD initiation and subsequent referral to treatment. Inclusion of methadone as a treatment option is particularly critical as the country continues to grapple with a surge in high potency synthetic opioid (HPSO) use, including fentanyl and fentanyl analogs.^29^ Patients using HPSOs have an increased risk of precipitated withdrawal during buprenorphine induction, thus creating a major barrier to buprenorphine initiation.^30^,^31^ Therefore, methadone will increasingly need to be considered as part of the treatment algorithm for those dependent on HPSOs.

Two recent studies reported that lack of familiarity with induction methods in the ED and time constraints were significant barriers to buprenorphine induction in the ED.^20^,^32^ This was not consistent with our study, which found that the main barriers to using either MOUD were a lack of access to follow-up addiction experts or social worker resources. The reason for this difference may be a result of both state and national education programs on buprenorphine utilization, as well as professional organization position statements, which led to the rapid acceptance and uptake of buprenorphine in the ED over the past few years.^33^ Emergency departments have worked to create community relationships with clinics that offer buprenorphine and these same relation-ships can be cultivated with local methadone clinics.^34^ Linkage to methadone clinics has been successful in the past when vouchers for methadone treatment were provided to patients discharged from the ED.^35^

Furthermore, published best practices for adopting buprenorphine programs in the ED can be adapted to facilitate ED-based methadone initiation. Examples include using a clinician champion to train colleagues and address administrative barriers and a trained substance-use patient navigator to facilitate linkage to outpatient methadone treatment and assist patients with social determinants of health-related barriers to long-term treatment engagement (eg, unreliable transportation).^36^ Additionally, a protocol for methadone dosing in the ED setting should be created, and all emergency clinicians should be trained in its use. A protocol may be particularly useful to overcome clinicians’ hesitation concerning methadone use in the EM setting, given its interindividual variability in pharmacokinetics and potential for dose accumulation.

Despite ED-based efforts to increase post-discharge treatment engagement, patients face pre-existing access challenges to receipt of methadone in the community. Unlike buprenorphine, which may be prescribed by any waivered clinician, methadone can only be dispensed at federally certified opioid treatment programs that require supervised daily on-site medication dosing. These regulations limit access and increase the stigma associated with methadone treatment for OUD.^37^ COVID-19 era regulations relaxed the requirements for on-site methadone dosing, allowing up to 28 days of take-home doses with no evidence of negative out-comes.^38^,^39^,^40^ As states move to retain relaxed methadone regulations beyond the pandemic, establishing pathways for linkage to outpatient methadone treatment will be even more critical.^41^

Given that the perceived barriers to buprenorphine and methadone initiation in the ED were similar in this study, we postulate that stigma may play a role in EPs’ choice of MOUD. Previous studies seem to support this theory and demonstrate clear bias against patients receiving methadone and the clinics that provide methadone.^20^,^42^ To combat the stigma of methadone initiation in the ED, the same tools used to reduce the stigma of treating patients with a SUD in other healthcare settings can be used. Interventions include the following: integrating MOUD training into medical school curriculums; having specialty addiction consult services in hospitals; and providing continuing education that focuses on increasing awareness of the benefits of MOUD and highlights the barriers to OUD treatment.^43^–^46^

In addition to increased education and structural support, more research must be completed to assess methadone initiation in the ED. Research topics include conducting basic epidemiological studies on methadone initiation in the ED, establishing and evaluating an ED methadone initiation protocol, and monitoring the rate of successful linkage to follow-up care after methadone initiation in the ED. Additional studies on interventions to reduce the stigma of methadone and other MOUD treatments among EPs should also be conducted.

## LIMITATIONS

This study had several limitations. The low response rate (18.4%) may have created some nonresponse bias in our results. This is similar to prior research reporting response rates of surveys distributed through a professional organization.^47^–^49^ Additionally, participants had to be members of ACEP to be invited to participate in this study. Response bias, specifically social desirability bias, could have led some EPs to select more supportive answers to adopting buprenorphine or methadone in the ED. Our study had a similar gender and race distribution of EPs in the US as reported elsewhere, although it is important to note that the majority of respondents identified as White males.^50^ Even though the demographic distribution of this study matches national patterns, they may not be generalizable to all EPs as the percentage of physicians who had completed their X-waiver in our study (38%) vastly differs from national estimates. A prior study reported that only 1% of EPs were X-waivered nationally.^51^ Another limitation of the study is that there was no way to control for multiple respondents from the same institution because this study’s survey and EMPRN did not collect institution-specific data. Lastly, it is important to note that our research team assumed that the EP had not ordered either MOUD if the participant did not answer the question of whether they had ordered buprenorphine or methadone while working in the ED.

## CONCLUSION

Our cross-sectional study demonstrates that, despite more than 50 years of data demonstrating methadone’s efficacy, emergency physicians are not comfortable using methadone for patients with opioid use disorder. Buprenorphine has been embraced by EPs, largely as a result of ongoing local, regional, and national education efforts, as well as widely publicized and distributed statements by influential professional organizations.^12^,^17^,^52^ Similar efforts should now be undertaken to educate and support emergtency physicians to increase methadone utilization and decrease the stigma frequently associated with this life-saving medication.

## Supplementary Information





## Figures and Tables

**Table 1:** Sociodemographic characteristics of emergency physicians with experience prescribing medications for opioid use disorder.

Variables	N	
Gender (N/%)	141	
Male		114 (80.9)
Female		27 (19.1)
Race (N/%)	137	
White		113 (82.5)
Black		1 (0.7)
Hispanic		1 (0.7)
Asian		5 (3.6)
Other races		17 (12.4)
Primary practice location (N/%)	140	
Rural		25 (17.9)
Suburban		53 (37.9)
Urban		62 (44.3)
Type of health system (N/%)	141	
Academic		45 (31.9)
Community		90 (63.8)
Federal		6 (4.3)
Years of experience (mean/SD)	114	20.4 (10.4)
Ever ordered MOUD in the ED (N/%)	141	
Buprenorphine		22 (15.6)
Methadone		20 (14.2)
Both		56 (39.7)
Neither*		43 (30.5)
Completed X-waiver (N/%)	140	
Yes		53 (37.9)
Department has bridge program to MOUD outpatient treatment (N/%)	141	
Yes, for buprenorphine		46 (32.6)
Yes, for methadone		1 (0.7)
Yes, for both methadone and buprenorphine		13 (9.2)
No		81 (57.4)
Location (N/%)	141	
Northeast		27 (19.1)
South		48 (34.0)
Midwest		34 (24.1)
West		32 (22.7)
Age (mean/SD)	141	53.4 (10.6)

* Neither was not an option on the survey. If participants didn’t answer this question, it was assumed they had never ordered buprenorphine or methadone in the ED.

*ED*, emergency department, *MOUD*, medication for opioid use disorder, *SD*, standard deviation.

**Table 2:** Experiences with prescribing medications for opioid use disorder vs primary practice location.

MOUD experiences	N	Rural	N	Suburban	N	Urban	*P*-value
Ordered MOUD in ED	25		53		62		
Buprenorphine		3 (12.0)		13 (24.5)		6 (9.7)	0.002
Methadone		2 (8.0)		11 (20.8)		7 (11.3)	
Both		5 (20.0)		18(34.0)		32 (51.6)	
Neither*		15 (60.0)		11 (20.8)		17 (27.4)	
Completed X-waiver	25		53		61**		
Yes		5 (20.0)		20 (37.7)		28 (46.0)	0.080
Department has warm handoff or bridge program to MOUD outpatient treatment	25		53		62		
Yes, for buprenorphine		4 (16.0)		17 (32.1)		24 (38.7)	0.152
Yes, for methadone		0 (0.0)		0 (0)		1 (1.6)	
Yes, for both		1 (4.0)		4 (7.5)		8 (12.9)	
No		20 (80.0)		32 (60.4)		29 (46.8)	

* ”Neither” was not an option on the survey.

** All questions were not required to be answered to participate in this study.

*MOUD*, medication for opioid use disorder, *ED*, emergency department.

**Table 3:** Highest perceived barrier and experience with medications for opioid use disorder by access to a bridge.

		Has bridge		No bridge	*P*-value
	N	n	N	n	
Highest ranked barriers for prescribing buprenorphine					
There is no reimbursement for me.	53	2 (3.8)	76	2 (2.6)	1.000
I don’t have access to providers for follow up in my area.	53	9 (17.0	78	14 (17.9)	0.886
There’s no financial incentive for my department.	52	3 (5.8)	76	5 (6.6)	1.000
It takes too much of my time.	53	8 (15.1)	78	7 (9.0)	0.280
I don’t have social work resources for screening and follow up.	54	4 (7.4)	77	21 (27.3)	0.004
I don’t have training.	53	3 (5.7)	77	12 (15.6)	0.082
I don’t have buprenorphine in my ED.	52	4 (7.7)	79	18 (22.8)	0.024
I don’t want to assume medicolegal risk.	54	1 (1.9)	78	6 (7.7)	0.239
Highest ranked barriers for dosing methadone					
There is no reimbursement for me.	53	1 (1.9)	66	5 (7.6)	0.224
I don’t have access to providers for follow-up in my area.	55	11 (20.0)	67	41 (61.2)	< 0.001
There’s no financial incentive for my department.	52	4 (7.7)	66	5 (7.6)	1.000
It takes too much of my time.	53	6 (11.3)	66	3 (4.5)	0.185
I don’t have social work resources for screening and follow-up.	51	2 (3.9)	67	14 (20.9)	0.008
I don’t have training.	53	2 (3.8)	67	12 (17.9)	0.017
I don’t have buprenorphine in my ED.	54	11 (20.4)	68	20 (29.4)	0.255
I don’t want to assume medicolegal risk.	54	8 (14.8)	67	13 (19.4)	0.508
Has ever prescribed	60		81		
Buprenorphine		12 (20.0)		10 (12.3)	<0.001
Methadone		6 (10.0)		14 (17.3)	
Both		36 (60.0)		20 (24.7)	
Neither*		6 (10.0)		37 (45.7)	

* ”Neither” was not an option on the survey

** All questions were not required to be answered to participate in this study.

*MOUD*, medication for opioid use disorder; *ED*, emergency department.
