# Supplementary material for: Attitudes on Methadone Utilization in the Emergency Department: A Physician Cross-sectional Study
Source: West J Emerg Med. 2022 Apr 4;23(3):386–95. doi: 10.5811/westjem.2022.2.54681 (PMC9183785; doi:10.5811/westjem.2022.2.54681)
Supplement: Supplementary file 2 [file wjem-23-386-s002.docx]

Perceptions of Methadone Dosing in the ED

- 1. What best describes your primary practice location? (Choose one answer)

Urban
Rural
Suburban

- 1. What best describes the type of health system you work in? (Choose one)

Community

Academic

Federal Government Hospital (Department of Defense, the Department of Health and Human Services, and the Veterans Health Administration)

1. About how many years have you been out of training?
2. Which of the following have you ever ordered while working in the ED? (Check all that apply)

Methadone
Buprenorphine

1. Have you obtained your X waiver to prescribe buprenorphine? (Choose one answer)

Yes
No

1. Does your department offer a warm handoff or a bridge program to outpatient treatment at discharge for ongoing methadone or buprenorphine treatment? (Choose one answer)

Yes, for methadone
Yes, for buprenorphine

Yes, for both methadone and buprenorphine

No

1. Please evaluate the following statements as they apply to the role of EM providers in addressing opioid use. (Strongly Agree, Somewhat Agree, Neutral, Somewhat Disagree, Strongly Disagree) (Choose one answer)
   1. EM providers should offer buprenorphine to help control the symptoms of opioid withdrawal and craving.
   2. EM providers should offer methadone to help control the symptoms of opioid withdrawal and craving.
   3. EM providers should refer patients they identify as having an opioid use disorder to a clinic that provides buprenorphine.
   4. EM providers should refer patients they identify as having an opioid use disorder to a clinic that provides methadone.
2. How much do you agree or disagree with the following statements? (Strongly Agree, Somewhat Agree, Neutral, Somewhat Disagree, Strongly Disagree)
   1. If my ED had a structured program, I would be comfortable starting buprenorphine for patients who are continuing it after discharge for the purpose of entering treatment.
   2. If my ED had a structured program, I would be comfortable dosing methadone for patients who are continuing it after discharge for the purpose of entering treatment.
   3. I'm concerned about patients returning to the ED for refills of buprenorphine.
   4. I'm concerned about patients returning to the ED for methadone dosing.
   5. Initiating patients on buprenorphine is not within the scope of an ED physician’s practice.
   6. Initiating patients on methadone is not within the scope of an ED physician’s practice.
3. How do you rate the following as barriers to you using buprenorphine in the ED? (1-biggest barrier, 8-very low or no barrier)
   1. There is no reimbursement for me.
   2. I don't have access to providers for follow up in my area.
   3. There's no financial incentive for my department.
   4. It takes too much of my time.
   5. I don't have social work resources for screening and follow up.
   6. I don't have training.
   7. I don't have buprenorphine in my ED.
   8. I don’t want to assume medicolegal risk.
4. How do you rate the following as barriers to you using methadone in the ED? (1-biggest barrier, 8-very low or no barrier)
   1. There is no reimbursement for me.
   2. I don't have access to providers for follow up in my area.
   3. There's no financial incentive for my department.
   4. It takes too much of my time.
   5. I don't have social work resources for screening and follow up.
   6. I don't have training.
   7. I don't have methadone in my ED.
   8. I don’t want to assume medicolegal risk.
